# Inborn Errors of Amino Acid Metabolism Revisited: Clinical Implications and Insights into Current Therapies

**DOI:** 10.3390/jcm14248749

**Published:** 2025-12-10

**Authors:** Abdul L. Shakerdi, Darragh Nerney, Eleanor J. Molloy, Ina Knerr

**Affiliations:** 1 School of Medicine, Trinity College Dublin, D02 PN40 Dublin, Ireland; shakerda@tcd.ie; 2 National Rare Diseases Office, Mater Misericordiae University Hospital, D07 R2WY Dublin, Ireland; darragh.nerney@ucdconnect.ie; 3 Discipline of Paediatrics, Trinity College Dublin, The University of Dublin, D02 PN40 Dublin, Ireland; eleanor.molloy@tcd.ie; 4 Neonatology, Children’s Health Ireland (CHI), D12 N512 Dublin, Ireland; 5 National Centre for Inherited Metabolic Disorders, Children’s Health Ireland (CHI), D01 XD99 Dublin, Ireland; 6 School of Medicine, University College Dublin, D04 V1W8 Dublin, Ireland

**Keywords:** amino acids, emergency therapy, inborn errors of metabolism, personalised medicine

## Abstract

**Background/Objectives:** Inborn errors of amino acid metabolism (IEAAMs) are inherited disorders caused by defects in amino acid catabolism, biosynthesis, or transport. In this review, we aimed to synthesise recent evidence on the clinical manifestations and current and future therapeutic strategies for major IEAAMs. **Methods:** A narrative review was undertaken on studies published up to November 2025. No fixed start date was set. Instead, earlier studies were included if historically significant or frequently cited in contemporary guidelines, and emphasis was placed on recent developments over the last 5–10 years. Evidence was identified through structured searches of PubMed, clinical trial registries, and public communications on selected IEAAMs, which were synthesised in textual and tabular form. **Results:** Management across IEAAMs involves the restriction of amino acids or natural proteins, disease-specific dietary formulations, micronutrient optimisation, cofactor or enzyme replacement, and pharmacological chaperones. This is supported by structured monitoring and emergency regimens to prevent catabolic crises. Organ transplantation remains crucial for select indications, such as liver transplantation in hereditary tyrosinaemia with liver disease. Novel approaches include substrate reduction, the pharmacological targeting of upstream pathways, viral vector gene transfer, and liver-directed mRNA therapy. Several of these novel approaches have entered clinical trials, but many remain in the preclinical stage. **Conclusions:** Despite advances in the treatment of IEAAMs, many patients still experience significant morbidity. Future focus should be on further refining emerging molecular and gene-based treatments and optimising neuroprotective and metabolic targets. The equitable implementation of personalised, life-spanning treatments within multidisciplinary rare disease services will be essential.

## 1. Introduction

Amino acids primarily serve as the fundamental building blocks of proteins. Some also play crucial roles in vital bodily functions or act as precursors for essential nitrogen-containing compounds including neurotransmitters, hormones, nucleotides, and pigments [1]. Humans use 21 distinct amino acids, the majority of which may be produced internally, but 9 (isoleucine, leucine, lysine, methionine, phenylalanine (Phe), threonine, tryptophan, valine, and histidine) are “essential” in the sense that they must be consumed through food [2].

The term “inborn errors of amino acid metabolism (IEAAMs)” refers to a wide range of metabolic disorders, including those brought on by enzyme deficiencies in the catabolic pathway of one or more amino acids or, less frequently, by cofactor or transporter deficiency. This may result in the toxic accumulation of certain amino acids or their respective catabolic intermediates, as well as the deficiency of key metabolic products. The result is a wide range of clinical manifestations and complications [3]. This review fills an important gap by providing a comprehensive and clinically focused update on recent advances in the diagnosis and management of the most prevalent IEAAMs.

IEAAMs include phenylketonuria (PKU), alkaptonuria (AKU), homocystinuria (HCU), maple syrup urine disease (MSUD), tyrosinaemia, pyridoxine-dependent epilepsy, glutaric aciduria type 1, and nonketotic hyperglycinaemia. The aim of treatment is to normalise the metabolic imbalance as much as possible. This can be achieved via dietary modification, pharmacological treatment, or in some instances cofactor supplementation. Key dietary interventions include amino acid-restricted diets or the restriction of natural protein with the restriction of offending amino acid(s), together with dietary supplementations of micronutrients and disease-specific amino acid supplements. Medications are used to reduce the formation of toxic by-products and enhance the removal of toxic metabolites in the body. For some conditions, pharmacotherapies are available that also include enzyme/cofactor replacement or pharmacological chaperones, enhancing residual enzyme activity or promoting alternative and accessory pathways. Continued patient follow-up may include clinical evaluation and the monitoring of potential long-term complications, surveillance of disease-specific biochemical indicators, medication management, dietetic modifications, and nutritional surveillance.

Emergency therapy may also be required in severe presentations and aims to promote anabolism, reduce catabolism, limit toxic metabolite formation, and enhance their clearance. General neuroprotective measures are also necessary in certain conditions and include the prevention of hypoglycaemia, early treatment of hyperammonaemia, and the reduction of hyperhomocysteinaemia. The prompt management of pyrexia/intercurrent infections and careful nutritional surveillance to avoid micronutrient deficiencies is crucial in some disorders. While existing therapeutic approaches must be optimised for better long-term results, clinical challenges remain and the disease burden is significant [3]. Novel treatments currently in development include mRNA and gene therapies, which hold the potential for more definitive or curative interventions. We performed a narrative review up to November 2025 through structured searches on PubMed, clinical trial registries, and publicly available communications. No fixed start date was set. Instead, earlier studies were included if historically significant or frequently cited in contemporary guidelines. An emphasis was placed on recent developments over the last 5–10 years. The information was then synthesised in both textual and tabular form for several IEAAMs. Table 1 provides a summary of the status and future directions of selected IEAAMs, which are examined in greater depth throughout the main sections of this review. We also present an overview of the management of some of the main IEAAMs outlined in tabular form. The medical and dietary management of the most commonly encountered disorders is also discussed in further detail.

## 2. Key Disorders of Amino Acid Metabolism: Clinical Features and Therapeutic Approaches

### 2.1. Phenylketonuria

**Figure 1:**
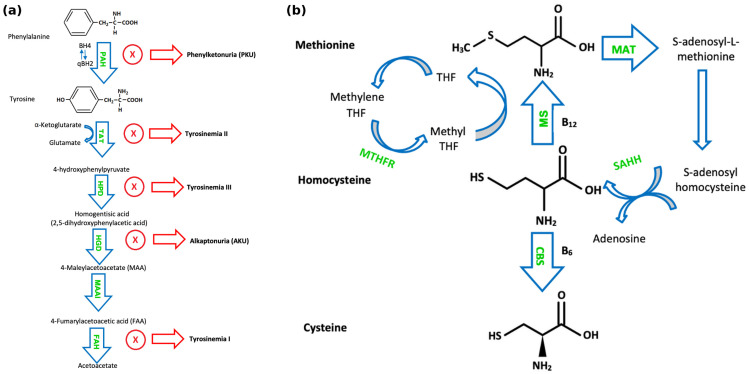
(**a**) Pathways of phenylalanine and tyrosine metabolism. “PAH” denotes phenylalanine hydroxylase; tyrosine aminotransferase (TAT); 4-hydroxyphenylpyruvate dioxygenase (HPD); homogentisate 1,2-dioxygenase (HGD); maleylacetoacetate isomerase (MAAI), which converts maleylacetoacetate (MAA) to fumarylacetoacetate (FAA); and fumarylacetoacetate hydrolase (FAH). (**b**) Pathways of sulphur-containing amino acid metabolism. “MS” denotes methionine synthase; cystathionine-β-synthase (CBS); tetrahydrofolate (THF); methylene tetrahydrofolate reductase (MTHFR); methionine S-adenosyltransferase (MAT); and S-adenosylhomocysteine hydrolase (SAHH).

Phenylketonuria (PKU) (OMIM #261600) is an autosomal recessive disorder characterised by an absence or profound deficiency of the enzyme Phe hydroxylase (PAH) (EC 1.14.16.1), which normally converts Phe to tyrosine (Tyr) (Figure 1a). Decreased PAH activity leads to the accumulation of Phe in the blood and brain [4]. It is usually detected through newborn bloodspot screening and subsequently confirmed with the detection of two pathogenic variants in the PAH gene. In PKU, accumulated Phe is converted to phenylpyruvic acid and phenyllactic acid, which are excreted in the urine. PKU has an incidence of ~1:16,000 live births in the US, with higher rates observed in countries such as Ireland and Turkey [5]. If untreated, PKU can result in intellectual disability, motor deficits, seizures, and eczema [6]. Blood samples for the analysis of Phe and Tyr should ideally be collected in the morning after overnight fasting. Dietary management remains the cornerstone of treatment for PKU for most paediatric patients. A practical dietary approach in PKU management involves restricting total natural protein intake (“protein exchanges”) rather than calculating individual amino acid contributions, permitting the free consumption of certain low-Phe fruits and vegetables based on defined thresholds. A compositional analysis of 165 fruits, vegetables, and starchy roots, published recently by our group, has expanded the evidence base for this approach [7]. The study confirmed that many fruits and vegetables contain Phe concentrations below the 75 mg/100 g threshold recommended by European PKU guidelines, and their unrestricted consumption does not adversely impact metabolic control. This is likely due to the lower digestibility of plant-based proteins and their high fibre content, which may reduce Phe bioavailability [7]. An evidence-based dietary liberalisation of certain foods in PKU has been proposed to enhance quality of life, dietary adherence, and nutritional adequacy.

The management of PKU consists of dietary Phe restriction through natural protein restriction, together with disease-specific amino acid supplements, including also Large Neutral Amino Acids (LNAAs) and, occasionally, additional Tyr supplementation. Biochemical monitoring is provided through blood testing for PKU patients. An upper blood Phe target level of 360 μmol/L is recommended for children with PKU aged < 12 years, and an upper target level of 600 μmol/L is recommended for older patients aged > 12 years (outside pregnancy) to achieve optimal outcomes [6]. Recent treatment developments for PKU include chaperone therapy and enzyme substitution therapy. Sapropterin dihydrochloride is a synthetic form of tetrahydrobiopterin (BH4), an essential cofactor for PAH activity which has been shown to reduce Phe concentrations in PKU patients [8]. In approximately 20–30% of PKU patients, Phe levels may be controlled by tetrahydrobiopterin (BH4) therapy [9]. A novel treatment option for PKU is pegvaliase, a pegylated derivative of Phe ammonia lyase (PAL), which converts Phe to ammonia and trans-cinnamic acid [10]. Ammonia and trans-cinnamic acid are subsequently metabolised in the liver and excreted in the urine, resulting in reduced Phe concentrations [10]. Sepiapterin is a synthetic precursor to BH4 that has been shown to result in a higher intracellular BH4 accumulation than sapropterin [11]. A recent phase III international clinical trial (NCT05099640) explored the role of sepiapterin in PKU. It was found that sepiapterin significantly reduced Phe concentrations by 63% compared with a placebo, with no serious adverse events in participants [12]. Sepiapterin was subsequently FDA-approved for the treatment of PKU in children and adults in July 2025 [13] (Table 2).

Ongoing research is investigating the efficacy of JNT-517, which inhibits the solute carrier family 6 member 19 (SLC6A19)-mediated reabsorption of Phe in the intestine and kidneys. In a phase I study on healthy volunteers, it was found to be safe and to increase the urinary excretion of Phe [14], and phase II trials in adolescent and adult PKU patients are ongoing [15,16]. Another investigational therapy is NGGT002, an AAV8-based gene therapy delivering a functional PAH gene, currently in clinical trials for adult patients in the US (phase I/II, NCT06332807) and China (phase I, NCT06061614) [17,18].

Recent preclinical research has identified that Phe produces its cognitive impairment effects at least partially through the activation of glutamate [NMDA] receptor subunit epsilon-2 (GluN2B)-containing NMDARs [19]. In addition, the pharmacological and viral suppression of GluN2B restored neuroplasticity and cognition impairment in a mouse model of PKU [19]. The inhibition of histone deacetylase 6, a protein involved in transcriptional regulation, has been shown to increase the expression of acetylated heat shock protein 90 (HSP90), and hence to interfere with the degradation of misfolded PAH and reduce plasma Phe levels [20]. These findings warrant further studies into these molecular targets aimed at improving Phe control and limiting the development of neurological complications in PKU.

**Table 2:** Medical and dietetic management of phenylalanine and tyrosine disorders.

Disorder	Treatment	Rationale/ Mechanism	Dose	Biochemical Monitoring
Phenylketonuria (PKU)	Phenylalanine-free amino acid supplements Dietary restriction of phenylalanine	To limit intake of offending amino acid	Small, frequent doses (3–4) spaced evenly across day [21].	Blood phenylalanine levels
	Sapropterin dihydrochloride (Kuvan^®^, San Rafael, CA, USA)	Synthetic form of cofactor tetrahydrobiopterin (BH4)	Recommended starting dose in patients is 10 mg/kg body weight/day. Dose is adjusted, usually between 5 and 20 mg/kg/day, to achieve and maintain blood Phe control [9,22,23].	Blood phenylalanine levels
	Pegvaliase (Palynziq^®^)	Recombinant phenylalanine ammonia lyase (PAL) enzyme (patients ≥ 16 years)	Recommended starting dose is 2.5 mg once per week for 4 weeks. Dose escalated gradually based on tolerability to daily maintenance dose needed to achieve blood Phe control. Maintenance dose is individualised to achieve blood Phe control [10,24,25].	Blood phenylalanine levels
	Sepiapterin (Sephience™)	Synthetic BH4 precursor	<6 months: 7.5 mg/kg; 6–12 months: 15 mg/kg; 1–2 years: 30 mg/kg; >2 years: 60 mg/kg [26].	Blood phenylalanine levels
Biopterin defects causing hyperphenylalaninaemia [27]	Dietary restriction of phenylalanine (GTPCH and DHPR deficiency patients); phenylalanine-free amino acid supplements	To limit intake of offending amino acid	Small, frequent doses (3–4) spaced evenly across day [21].	Blood phenylalanine levels
	Sapropterin dihydrochloride (Kuvan^®^)	Synthetic form of cofactor tetrahydrobiopterin (BH4)	Recommended starting dose in adult patients is 10 mg/kg body weight/day. Dose is adjusted, usually between 5 and 20 mg/kg/day, to achieve and maintain blood phenylalanine control.	Blood phenylalanine levels
	l-3,4- dihydroxyphenylalanine/carbidopa (L-DOPA) and 5-OH-Tryptophan	For neurotransmitter related movement disorders	L-DOPA in four divided doses with similar dosing for 5-OH-Tryptophan [28]; age-dependent.	LP for CSF neurotransmitters measurement (HVA, 5-HIAA); prolactin levels
	Folinic acid	For movement disorders, to prevent cerebral folate deficiency	Dose of 10–15 mg/day [28].	Monitoring of CSF folate and folinic acid status
Hyperphenylalaninaemia due to DNAJC12	Dietary restriction of phenylalanine; phenylalanine-free amino acid supplements	To limit intake of offending amino acid	Small, frequent doses (3–4) spaced evenly across day [21].	Blood phenylalanine levels
	Sapropterin dihydrochloride (Kuvan^®^)	Synthetic form of cofactor tetrahydrobiopterin (BH4)	Recommended starting dose in adult patients is 10 mg/kg body weight/day. Dose is adjusted, usually between 5 and 20 mg/kg/day, to achieve and maintain blood Phe control.	Blood phenylalanine levels
	L-DOPA and tryptophan	For neurotransmitter related movement disorder	Starting dose of 2.5 mg/kg/day (can be increased to 6 mg/kg/day) [29].	LP for CSF neurotransmitters measurement (HVA, 5-HIAA)
Alkaptonuria (AKU)	Dietary restriction of phenylalanine; tyrosine/phenylalanine-free amino acid supplements	To limit intake of offending amino acid	Moderate restriction of natural protein.	Plasma amino acids (phenylalanine, tyrosine)
	Nitisinone (currently nitisinone is approved for alkaptonuria treatment in adults only)	Inhibits 4- hydroxyphenylpyruvate dioxygenase	The recommended dose in the adult AKU population is 10 mg once daily [30,31].	Plasma amino acids (phenylalanine, tyrosine)
	Bisphosphonate [32]	Inhibit bone resorption by preventing hydroxyapatite breakdown	As clinically indicated.	Bone turnover markers (BTMs)
	Teriparatide [32]	Promotes bone anabolism through protein kinase A and protein kinase C pathways [33]	Dose of 20 mcg/day SC (approved in adults).	BTMs, plasma calcium levels
Tyrosinaemia type I	Dietary restriction of phenylalanine and tyrosine; tyrosine/phenylalanine-free amino acid supplements	To limit intake of offending amino acids		Plasma amino acids (phenylalanine, tyrosine, methionine), liver function, blood/urine succinylacetone
	Nitisinone (nitisinone is approved for tyrosinaemia type I treatment in children)	Inhibits 4- hydroxyphenylpyruvate dioxygenase	Recommended starting dose in adult patients is 1 mg/kg body weight/day. Dose should be adjusted individually. Maximum of dose of 2 mg/kg body weight/day [34,35].	Blood tyrosine levels, blood/urine succinylacetone, NTBC drug levels, liver function, alpha-fetoprotein
	Liver transplant	If end-stage liver disease, liver failure, or hepatocellular carcinoma develops		
Tyrosinaemia type II	Dietary restriction of phenylalanine and tyrosine; tyrosine/phenylalanine-free amino acid supplements	To limit intake of offending amino acids		Blood tyrosine and phenylalanine levels
Tyrosinaemia type III	A restrictive tyrosine and phenylalanine diet has been suggested during childhood [20], while other authors argue that such restriction is not recommended			

### 2.2. Alkaptonuria

Alkaptonuria (AKU) (OMIM #203500) is caused by mutations in HGD (OMIM 607474), leading to a homogentisic acid oxidase (EC 1.13.11.5) deficiency and consequent homogentisic acid (HGA) accumulation (Figure 1a). AKU has an autosomal recessive inheritance pattern, with a global prevalence of 1:250,000–1:1,000,000 [36]. Accumulated HGA is oxidised to benzoquinone acetate which subsequently forms melanin-like polymers. These polymers accumulate in collagen in a process called ochronosis. Many affected individuals are asymptomatic until adulthood. A typical feature of AKU is the darkening of urine upon standing for a period of time, reflecting homogentisic aciduria. Ochronotic pigment accumulation in the connective tissues of various organs may lead to grey pigmentation of the sclera and ear helix, ochronotic osteoarthropathy, nephrolithiasis, cholelithiasis, prostatic calculi, and valvular dysfunction [37]. AKU is also associated with increased levels of serum amyloid A and the development of secondary amyloidosis, a complication proposed to result from chronic inflammation and oxidative stress [38]. The treatment of AKU in childhood involves a moderate protein restriction, hence limiting the Phe and Tyr intake to physiological requirements; Tyr/Phe-free amino acid supplements can be used to enhance dietary efficacy [39]. Some centres also suggest ascorbic acid at a dose of 250–500 mg/day. A pharmacological option is nitisinone 2-(2-nitro-4-trifluoromethylbenzyl)-1,3-cyclohexanedione (NTBC), which inhibits 4-hydroxyphenylpyruvate dioxygenase (HPD) and hence reduces serum and urinary HGA levels, which may reverse or slow the rate of the development of complications associated with alkaptonuria [30] (Table 2). Patients require clinical surveillance, e.g., from an orthopaedic, cardiac, renal, ophthalmic, and neurological viewpoint. Recent research in the space of AKU has identified the SAA1.1 allele as a genetic determinant and potential biomarker for inflammation and AA amyloidosis in AKU patients [40]. However, further clinical validation is necessary. To our knowledge, there are no ongoing clinical trials for novel therapies in AKU.

### 2.3. Tyrosinaemia Type I, Type II, and Type III

Tyrosinaemia type I (HT1, OMIM #276700) is an inborn error of Tyr catabolism with autosomal recessive inheritance. The primary enzyme defect is a deficiency of fumarylacetoacetate hydrolase (FAH, EC 3.7.1.2). The birth incidence is 1/100,000 in most regions, with higher rates in Scandinavia and Québec, Canada [35]. It often presents acutely before the age of 2 months with the acute failure of hepatic synthetic function, hepatomegaly, and coagulopathy. The sub-acute and chronic forms present after 2 months and 6 months, respectively, typically displaying a lesser degree of hepatic impairment, but with the additional burden of proximal renal tubulopathy resulting in renal tubular acidosis, aminoaciduria, hypophosphataemic rickets, and a failure to thrive [41]. Porphyria-like syndromes may also occur [42]. Patients have a significantly increased risk of developing hepatocellular carcinoma [41]. Early treatment with NTBC and dietary Phe and Tyr restriction is thus crucial to limit the development of these complications. Liver transplant may be necessary in patients with hepatocellular carcinoma or NTBC-refractory decompensated hepatic failure [43] (Table 3). The therapeutic administration of a lentiviral vector provided stable long-term FAH expression in porcine HT1 and a reduction in the development of both premalignant and malignant lesions [44]. The genetic deletion of HPD has also been proposed as a long-term treatment of the disease. Paradoxically, a recent study in a mouse model of HT1 showed that, while the AAV8 CRISPR deletion of HPD results in metabolic correction and phenotypic rescue, it resulted in a marked increase in hepatocellular carcinoma [45]. This may have important implications for future clinical trials and the development of gene-editing therapies, underscoring the need for rigorous long-term safety monitoring, particularly in disorders with an existing predisposition to cancer. Another promising avenue of research is the use of an engineered Escherichia coli strain that has been shown to degrade Tyr, reduce the accumulation of toxic metabolites, and prevent hepatic injury in a HT1 mouse model [46].

Tyrosinaemia type II (OMIM #276600) is caused by a defect in the first step of Tyr degradation catalysed by Tyr aminotransferase (TAT) (EC 2.6.1.5), resulting in the accumulation of Tyr. It has an incidence of less than 1 in 250,000 [47]. It may present with scleral inflammation, pseudoherpetiform corneal ulceration, and palmoplantar hyperkeratosis, in addition to neurological symptoms, mostly in the form of intellectual disability. Treatment is with a protein-restricted diet low in Tyr and Phe [48]. Oculocutaneous lesions may subside with dietetic treatment, but not the neurological features [49] (Table 2).

Tyrosinaemia type III (OMIM #276710) is caused by an impairment in the HPD (EC 1.13.11.27) enzyme, which is one step downstream of TAT [50]. Less than 20 cases have been reported [51]. Tyrosinaemia type III is characterised by elevated plasma levels of Tyr and an increased urinary excretion of 4-hydroxyphenylpyruvate, 4-hydroxyphenyllactic acid, and 4-hydroxyphenylacetate [52] (Figure 1a). Clinical features include ataxia, seizures, and developmental issues [53]. Tyrosinaemia type III patients do not usually demonstrate hepatorenal dysfunction or skin or eye lesions. A low natural protein diet, restrictive in Tyr and Phe, has been suggested during childhood for this ultra-rare condition [54], while other authors argue that such restriction is not recommended, or at least not necessary, as they present a case report of an asymptomatic 11-year-old girl despite no dietary modification [53] (Table 2).

### 2.4. Homocystinuria

The most common cause of inherited homocystinuria is cystathionine beta-synthase (CBS) (EC 4.2.1.22) deficiency, causing classical homocystinuria (HCU) (OMIM #236200), a disorder of methionine and homocysteine metabolism. HCU has an autosomal recessive inheritance. The worldwide prevalence of classical HCU is estimated to be 0.82:100,000; it is estimated to affect approximately 1 in 100,000–200,000 people in the United States [55,56]. CBS is a pyridoxine-dependent enzyme that converts homocysteine to cystathionine in the transsulfuration pathway (Figure 1b). Another pathway for homocysteine metabolism is its remethylation to methionine by the enzyme methionine synthase, using the folate derivative, methyltetrahydrofolate, as a methyl donor. Methionine synthase (EC 2.1.1.13) is a cobalamin-dependent enzyme [57]. A deficiency in methylenetetrahydrofolate reductase (EC 1.5.1.20), which catalyses the reduction of methylenetetrahydrofolate to 5-methyl-tetrahydrofolate, can result in moderate homocystinuria [57,58]. HCU can affect several organs, leading to osteopaenia, cognitive impairment, optic lens subluxation, and an increased risk of thromboembolism [57]. HCU treatment aims to maintain total plasma homocysteine (tHcy) levels < 50 μmol/L in pyridoxine-responsive patients and <100 μmol/L in non-pyridoxine-responsive patients to prevent comorbidities [59]. Elevated methionine levels can also be associated with significant morbidity in HCU. In a cohort study of 36 Irish infants with classical HCU, one case of hypermethioninemic encephalopathy was observed, associated with a plasma methionine level of 1329 μmol/L [60]. The treatment of HCU includes a low-protein diet and low-methionine formula, folate, cobalamin (vitamin B12) as needed, pyridoxine (vitamin B6), and betaine medication. Patient responsiveness to pyridoxine is assessed by prescribing 10 mg/kg/day (max. 500 mg), with the monitoring of plasma tHcys and methionine levels over the course of a few days. Patients whose plasma tHcy levels decrease below 50 μmol/L are classed as pyridoxine-responsive and do not require additional treatment [59]. It is important to note that long-term high-dose pyridoxine can cause peripheral neuropathy [61]. Betaine is best used as an adjunctive treatment for patients who are unable to achieve an adequate tHcy control by other methods. It can be initially prescribed at 3 g twice a day and may be increased to up to 150–200 mg/kg/day [59] (Table 3). It is generally important to ensure that children with HCU are well hydrated, especially during periods of increased metabolic stress and intercurrent illnesses.

Pegtibatinase is a human PEGylated CBS being developed as an enzyme-replacement therapy for HCU. In the COMPOSE phase I/II trial in patients aged 12–65, it was shown to be generally well-tolerated and effective at reducing total plasma homocysteine levels [62]. The therapy progressed to the HARMONY phase III pivotal trial, which was paused due to production scale-up problems. The manufacturing company says they are engaging with the FDA to recommence enrolment in 2026 [63].

In the preclinical space, CDX-6512/SYNT-202 is a promising therapeutic agent, acting as a gastrointestinal-stable methionine gamma-lyase enzyme to break down dietary methionine, and hence reduce substrate flow into the homocysteine pool. It was shown to reduce both methionine and homocysteine levels in HCU mice [64], pending clinical trials. An interesting approach to biochemical rescue in HCU is the use of FDA-approved proteasome inhibitors bortezomib and carfilzomib, which inhibit the degradation of misfolded CBS and induce certain HSPs [65]. Unfortunately, however, these therapies are likely to be too toxic for long-term patient treatment, particularly haematological and immunological adverse effects. Other therapies that have been investigated in early preclinical research include AAVrh.10-CBS gene therapy [66] and minicircle-based gene therapy [67].

**Table 3:** Medical and dietetic management of *Sulphur*-containing amino acid disorders.

Disorder	Treatment	Rationale/Mechanism	Dose	Monitoring
Homocystinuria (HCU) due to cystathionine beta-synthase (CBS) deficiency	Methionine-free amino acid supplements; dietary restriction of methionine/proteind supplementation of cysteine, B12, folate	To limit intake of offending amino acid	Individualised to patient	Methionine and cystine levels, B12, folate
	Pyridoxine (vitamin B6) (in pyridoxine-responsive patients)	Cofactor of cystathionine β-synthase	Recommended dose of up to 10 mg/kg/day; recommended to avoid doses >500 mg/day (risk of peripheral neuropathy) [59].	Plasma tHcy
	Betaine	Betaine donates a methyl group via betaine homocysteine methyl transferase (BHMT)	Recommended starting dose of 3 g BD; can increase up to 200 mg/kg/day; rarely benefits from higher dose [59]	Plasma tHcy
Homocystinuria due to methylene tetrahydrofolate reductase deficiency	Betaine	Betaine donates a methyl group via betaine homocysteine methyl transferase (BHMT)	Recommended starting dose of 3 g BD; can increase up to 200 mg/kg/day; rarely benefits from higher dose [59]	Plasma tHcy
	Aspirin	Antiplatelet therapy post-stroke	40.5 mg per second day [68]	Routine monitoring not recommended
	Supplementation of creatine, B6, B12, folate, 5MTHF	To achieve target plasma tHcy levels	Creatine (75–100 mg/kg/day), B6 (25 mg/day), B12 (25 mg/day), folate (4 mg/day), 5MTHF (2.4–3.2 mg/day) [68]	Creatinine, B6, B12, folate, 5MTHF levels
Methionine S-adenosyltransferase deficiency	S-adenosyl-L-methionine disulfate tosylate (SAM) supplementation	For neurological manifestations	400–800 mg BD [69]	SAM concentration in plasma and CSF
	Methionine-free amino acid supplements; dietary restriction of methionine/protein	To limit intake of offending amino acid (although may decrease S-adenosyl-L-methionine (SAM) synthesis [70])	Individualised to patient	Methionine levels
S-adenosylhomocysteine hydrolase deficiency	Methionine-free amino acid supplements; dietary restriction of methionine/protein	To limit intake of offending amino acids; to reduce toxic SAH levels	Individualised to patient	Methionine levels
	Phosphatidylcholine and creatine supplementation	Low levels of creatine and choline in SAH hydrolase deficiency	Creatine—e.g., 375 mg/kg/d Phosphatidylcholine—e.g., 150 mg/kg/d [71]	Creatinine, choline levels; blood/urine creatine
Cystinosis	Cysteamine	Depletes lysosomal cystine levels	1.30 g/m^2^/day; maximum of 1.95 g/m^2^/day [72]	WBC cystine assay
	Symptomatic treatment	Management of symptoms	E.g., ACE inhibitors for proteinuria; kidney transplant in ESRD; HRT for endocrinopathies	Depends on symptoms

### 2.5. Methylmalonic Acidaemia

Methylmalonic acidaemia (MMA; OMIM #251000 for MMUT, #251100 for MMAA, #251110 for MMAB) is a genetically heterogeneous group of autosomal recessive IEAAMs characterised by elevated methylmalonic acid in plasma and urine due to the impaired isomerisation of methylmalonyl-CoA to succinyl-CoA in mitochondrial propionate metabolism in the pathway of isoleucine, methionine, threonine, and valine [73]. The most common cause is a complete or partial deficiency of methylmalonyl-CoA mutase (EC 5.4.99.2) or defects in its cofactor 5′-deoxyadenosylcobalamin synthesis or transport. MMA has an incidence that varies globally, ranging from 1:50,000 to 1:360,000 [74]. Biochemically, affected individuals present with markedly elevated plasma MMA, increased urine MMA, hyperammonaemia, metabolic ketoacidosis, and elevated C3 acylcarnitine on newborn screening [75]. Neonates present with lethargy, vomiting, respiratory distress, and encephalopathy, which can progress to coma if untreated [76]. Children may also show a failure to thrive, developmental delay, and renal impairment [77]. Diagnosis is established via molecular testing for biallelic pathogenic variants or enzyme assays [75]. Management includes a dietary protein restriction particularly of propiogenic amino acids, high-calorie intake, carnitine supplementation, and hydroxocobalamin in B12-responsive forms, with liver and/or kidney transplantation considered in severe cases [75,78]. Carglumic acid is approved by the FDA as an adjunctive treatment for acute hyperammonaemia [79]. Metronidazole can be used to reduce propionate production by the gut flora. Despite early treatment, long-term complications such as chronic renal failure, basal ganglia injury, pancreatitis, and intellectual disability are common [75,80,81]. Children will require an individualised “sick day” plan to use when they are unwell, including the further restriction of natural protein, providing dextrose and fat as metabolic substrates, and increasing medications, such as carnitine and vitamin B12.

For mut-type MMA, the current front-runner in novel therapeutics is mRNA-3705, encoding MMUT formulated within a lipid nanoparticle. It showed 2.1–3.4-fold higher levels of hepatic MMUT protein expression and a greater reduction in plasma methylmalonic acid than an older generation mRNA therapy [82]. A phase I/II study (NCT04899310) aimed at assessing the safety, pharmacokinetics, and pharmacodynamics of mRNA-3705 in children and adults with mut-type MMA is currently ongoing [83].

### 2.6. Maple Syrup Urine Disease

**Figure 2:**
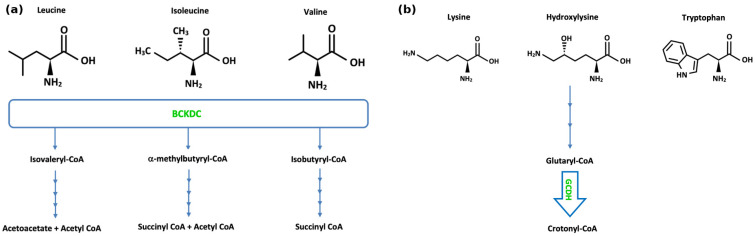
(**a**) Pathways of branched-chain amino acid metabolism. “BCKDC” denotes branched-chain α-ketoacid dehydrogenase complex. (**b**) Pathways of lysine, hydroxylysine, and tryptophan metabolism. “GCDH” denotes glutaryl-CoA dehydrogenase.

MSUD (OMIM #248600) is inherited in an autosomal recessive manner, with an incidence of approximately 1 in 185,000 people. MSUD is caused by pathogenic variants in the underlying genes BCKDHA (OMIM #608348), BCKDHB (OMIM #248611), or DBT (OMIM #248610), which abrogate the function of branched-chain ketoacid dehydrogenase (BCKDH), resulting in a partial or complete lack of branched-chain ketoacid dehydrogenase (BCKD) complex (EC 1.2.4.4) (Figure 2a). Symptoms of acute intoxication include poor feeding, vomiting, irritability, neuropsychiatric symptoms, lethargy, abnormal tone or movements, seizures, ataxia, and coma. Controlling several factors that affect endogenous protein anabolism and catabolism, plasma amino acid concentrations, and plasma osmolarity is necessary for the effective management of the complex pathophysiology of MSUD [84]. The intake of branched-chain amino acids (BCAAs) must be strictly limited in patients with MSUD, which is achievable in a diet low in natural protein and leucine, using medical foods and synthetic formulas containing micronutrients, such as vitamins, minerals, and essential fats, with prescribed amounts of isoleucine and valine supplementations. An additional thiamine challenge of 150–300 mg per day over one month followed by the evaluation of plasma BCAA levels is useful to identify patients who are thiamine-responsive, likely possessing residual BCKD activity, and hence would benefit from continued thiamine supplementation [85]. Acute crisis in MSUD is a medical emergency which may result in adverse or even fatal outcomes. The goal in acute management is to lower toxic levels of BCAAs, particularly leucine, and thereby their corresponding ketoacids in blood and other bodily fluids, and to reverse catabolism and promote anabolism. Management involves treating the underlying cause of the metabolic decompensation, ceasing natural protein intake, e.g., for 24 h with an increased intake of MSUD-specific formulas, providing hydration and extra “unwell” calories through carbohydrates and fat (PO/NG or IV), and correcting any metabolic abnormalities. Caloric support through IV dextrose infusion (e.g., 0.9% NaCl with 10% dextrose at 1.2–1.5 times maintenance, with potassium added as required) should be initiated as soon as possible. The use of BCAA-free formula in combination with valine and isoleucine supplementation can further promote anabolism and reduce plasma leucine levels, and, together with careful fluid and electrolyte management, prevent the development of cerebral oedema and brain injury [86,87]. In serious cases, more forceful detoxifying approaches may be taken, e.g., haemofiltration/haemodialysis (Table 4). In our study of 18 patients diagnosed with MSUD on newborn bloodspot screening in Ireland between 1972 and 2020, it was found that, despite early diagnosis and intervention, 12 patients required some form of dialysis during childhood, 6 of whom required dialysis in the neonatal period. Haemodialysis was found to be significantly more effective than peritoneal dialysis in lowering plasma leucine concentrations, with a ~28-fold faster reduction rate [88].

MSUD may be a candidate for gene therapy as a potential treatment option. Pontoizeau et al. [89] evaluated the treatment of severe MSUD in BCKDHB-knockout mice using an adeno-associated virus 8 vector carrying the human BCKDHB gene under the control of the ubiquitous human elongation factor 1-alpha promoter. This gene therapy provided long-term phenotypic rescue in the treated mice and reduced BCAA accumulation. Translating this success into human studies, particularly in a neonatal setting, may pose various difficulties, partly due to the age-related decline in liver trans-gene expression in humans, as well as the decreased efficiency of AAV8 in transducing human tissue compared with mice. In another study, the AAV9 dual-gene delivery of both BCKDHA and BCKDHB prevented perinatal death, normalised growth, and improved disease biomarkers in mice despite a high protein intake [90].

Recent research investigated IV lipid nanoparticles encapsulating chemically modified mRNAs encoding the three human BCKDH subunits (BCKDHA, BCKDHB, DBT) in mouse models of MSUD [91]. The therapy produced dose-dependent improvements in survival, with associated reductions in serum leucine levels in an intermediate (DBT-hypomorphic) MSUD model and extended survival with increased body weight in DBT-KO and BCKDHA-KO models, but showed no survival benefit in the BCKDHB-KO model [91].

**Table 4:** Medical and dietetic management of branched-chain amino acid disorders.

Disorder	Treatment	Rationale/Mechanism	Dose	Monitoring
Maple syrup urine disease (MSUD)	Synthetic formula with all amino acids except leucine, isoleucine, valine; valine and isoleucine supplementation; protein-free foods	To limit intake of offending amino acids	Valine: 15–30 mg/kg Isoleucine: 10–30 mg/kg [92], individualised to patient	Plasma levels of BCAAs
	Thiamine (vitamin B1) (in thiamine-responsive patients)	Increases stability of branched-chain alpha-ketoacid dehydrogenase complex (BCKDC)	Additional thiamine challenge of 150–300 mg/day for one month; continue thiamine supplementation in responsive patients [85]	Plasma levels of BCAAs
	Liver transplantation	Hepatic enzyme replacement		
	Management of acute crises: BCAA-free formula (PO or NG if not tolerating formula); provide all amino acids except leucine; supplement isoleucine and valine [37]. Reverse catabolism: increase calorie intake—IV calories (typically dextrose at high concentration); may start insulin drip if hyperglycaemic; use of normal or hypertonic saline; avoid hypotonic solutions; mannitol; diuretics; haemodialysis/haemofiltration.			
Methylmalonic acidaemia	Protein-restricted diet using synthetic propiogenic-devoid formulas	Reduce MMA production		Urine MMA, plasma amino acid concentrations
	Hydroxocobalamin	Enhance activity of methylmalonyl-CoA mutase	1 mg intramuscularly, regular continuation depends on metabolic response [76]	Urine MMA, plasma amino acid concentrations
	Carnitine	To correct secondary carnitine deficiency	50–100 mg/kg/day and up to ~300 mg/kg/day divided into 3–4 doses [76]	Plasma free carnitine level, acylcarnitine profile in dried blood spots
	Metronidazole	Reduce propionate production by gut flora	10–15 mg/kg/day typically administered in 7–10 day courses every 1–3 months [76]	Urine MMA, propionylcarnitine

### 2.7. Nonketotic Hyperglycinaemia

Nonketotic hyperglycinaemia (NKH) (OMIM #605899) is an autosomal recessive inherited disorder of glycine metabolism due to mutations in GLDC (OMIM #238300) or AMT (OMIM #238310), encoding the P- and T-proteins, respectively, which results in diminished or absent activity of the glycine cleavage enzyme system [93]. Consequently, glycine accumulates in tissues, particularly the central nervous system (CNS). Diagnosis is established with elevated plasma and CSF glycine, brain imaging, and most often confirmed by molecular genetics [94]. Preliminary research is also investigating the potential utility of miRNA-based biomarkers for the earlier and non-invasive diagnosis of NKH [95]. High levels of glycine may overstimulate N-methyl-D-aspartate (NMDA) receptors and can impact CSF serine and threonine levels. NKH has an incidence of 1:76,000 live births [93]. NKH is phenotypically divided into a severe form or an attenuated form. Most patients present with the severe form of NKH, which manifests with epileptic encephalopathy, spasticity, and psychomotor developmental delay. Seizures in patients with the attenuated form of NKH are usually readily manageable, and these patients display varying levels of developmental progress [96]. A glycine-restricted diet alone is insufficient for achieving a therapeutic effect [97]. The main goal of treatment is to reduce brain glycine levels to limit the impact of glycine as an NMDA receptor co-agonist. Sodium benzoate may be used, as it conjugates with glycine to form hippurate. Plasma concentrations of glycine should be regularly monitored to minimise the development of adverse events [98]. A ketogenic diet is a nonpharmacological option which has been proposed to reduce glycine levels and improve seizure control, particularly in severe disease. It may improve muscle tone, increase alertness, and reduce spasticity [98]. Dextromethorphan, an inhibitor of NMDA receptors, is also used. However, a recent critical narrative reassessment argued that routine NMDA antagonist therapy (dextromethorphan or ketamine) remains insufficiently evidence-based. It notes that studies lack controlled designs and clear benefits beyond glycine-lowering strategies [99]. Future controlled trials with objective endpoints and careful safety evaluation are needed before the routine endorsement of these therapies. Pharmacogenetics is also an important consideration in dextromethorphan therapy, as it is metabolised by the highly polymorphic CYP2D6 [100]. This genetic variability may result in various phenotypic extremes, including poor metabolisers and ultrarapid metabolisers, impacting drug efficacy and toxicity (Table 5).

As with many IEAAMs, gene therapy is under preclinical investigation in NKH. AAV9-GLDC treatment in mouse models has been shown to significantly reduce plasma and brain tissue glycine and normalise the folate profile, reflecting the recovery of glycine-derived one-carbon supply [101].

**Table 5:** Medical and dietetic management of lysine, serine, and glycine metabolism.

Disorder	Treatment	Rationale/Mechanism	Dose	Monitoring
Nonketotic hyperglycinemia (NKH)	Sodium benzoate Ketogenic diet (high in fat and low in carbohydrates) in some cases	Glycine reduction Alternate energy source for brain, epilepsy treatment with clobazam/ multidrug regimen		Glycine Blood glucose and ketones
	Sodium benzoate	Forms conjugated metabolite (hippurate), which is excreted by kidneys	Attenuated NKH—200–550 mg/kg/day Severe NKH—550 (–750) mg/kg/day (maximum dose 16.5 g/m^2^/day) [102]	Glycine in plasma and CSF
	Dextromethorphan (gene–drug interactions: CYP2D6, CYP3A4, CYPUGT)	Weak, non-competitive inhibitor of NMDA receptors	3–15 mg/kg/day (high individual variability) [27]	Glycine in plasma and CSF
	Pyridoxal phosphate (active form of vitamin B6)	Cofactor of glycine decarboxylase (GLDC)		Glycine in plasma and CSF
PDE-ALDH7A1	Pyridoxine (vitamin B6)	Pyridoxal 5′-phosphate (PLP) is a cofactor of enzymatic reactions involved in neurotransmitter synthesis	Adults: 200–500 mg/day (maximum dose 500 mg/day) [103]	Serum/plasma pipecolic acid levels, alpha-aminoadipic semialdehyde [AASA] in serum/plasma, urine, or CSF
	Lysine reduction therapies (LRTs)—lysine restriction, arginine supplementation	Arginine is a competitive inhibitor of lysine transport	Start at 4 g/m^2^/day (Maximum dose 5.5 g/m^2^/day) [103]	Plasma lysine, arginine
3-Phosphoglycerate dehydrogenase deficiency	L-Serine and glycine	Seizure control, correction of behavioural abnormalities	Infantile 3-PGDH deficiency: 500–700 mg L-serine/kg/d and 200– 300 mg glycine/kg/d Juvenile 3-PGDH deficiency: 100–150 mg L-serine/kg/d [104]	CSF serine and glycine; plasma serine and glycine
Phosphoserine aminotransferase deficiency	L-Serine and glycine	Prevention of neurological abnormalities in presymptomatic patients	L-serine: 500 mg/kg/day Glycine: 200 mg/kg/day [105]	CSF serine and glycine; plasma serine and glycine
3-Phosphoserine phosphatase deficiency	L-Serine	May prevent onset of neurological symptoms	200–300 mg/kg/day [105]	CSF and plasma serine

### 2.8. Pyridoxine-Dependent Epilepsy

Pyridoxine-dependent epilepsy (PDE) is an inborn error of lysine catabolism. The phenotype of PDE results from multiple genetic disorders [106]. Known genetic causes of PDE are biallelic pathogenic variants in any of three genes: PNPO (OMIM #603287), which encodes pyridox(am)ine 5′-phosphate oxidase (EC 1.4.3.5), PLPBP (OMIM #604436), which encodes PLP homeostasis protein, and ALDH7A1 (OMIM #107323), encoding the enzyme α-aminoadipic acid semialdehyde (α-AASA) dehydrogenase. PDE-ALDH7A1 (OMIM #266100) is the most common of the three and results from an autosomal recessive inherited deficiency of α-AASA. The incidence of PDE-ALDH7A1 is around 1:65,000 live births [107]. Deficient enzyme activity in the pipecolic acid and saccharopine catabolic pathway of lysine results in the abnormal accumulation of pipecolic acid, α-AASA, and Δ1-piperideine-6-carboxylate (Δ1-P6C) [106].

PDE is characterised by recurrent seizures that are resistant to conventional anti-epileptic drugs but can be effectively managed with pyridoxine (vitamin B6) supplementation. Elevated levels of α-AASA and pipecolic acid in a patient with an epileptic encephalopathy would suggest a diagnosis of PDE-ALDH7A1. Genetic testing confirms the diagnosis. A rapid and dramatic improvement in seizure control upon the administration of vitamin B6 strongly suggests PDE. The cornerstone of PDE treatment is pyridoxine supplementation, which can be administered orally or intravenously. In most cases, patients with PDE will require lifelong pyridoxine supplementation to control their seizures. The regular monitoring of blood pyridoxal 5′-phosphate (PLP) levels is necessary to ensure that patients are receiving an adequate dosage of vitamin B6. Maintaining PLP levels within the therapeutic range is crucial for effective seizure control. In some cases, patients with PDE may require anti-epileptic drugs (AEDs) in combination with pyridoxine. Despite seizure control, a large percentage of affected individuals (75%) may have developmental issues or a reduced intellectual ability [107] (Table 5).

A recent study investigated the targeting of the upstream 2-aminoadipic semialdehyde synthase (AASS) enzyme as a therapeutic strategy in PDE. This first mammalian proof-of-principle study showed that the knockout of AASS in PDE mice (double-knockout AASS/ALDH7A1) decreased levels of metabolites suspected to be neurotoxic, such as Δ1-piperideine-6-carboxylate and pipecolic acid in brain and liver tissues [108]. It also reduced the plasma levels of known PDE biomarkers [108]. As a similar concept, the genetic perturbation of lysine α-ketoglutarate reductase has also been shown to reduce the accumulation of toxic lysine metabolites, attenuate the epileptic phenotype, and improve developmental outcomes in ALDH7A1-deficient mice [70]. While still in the very early stages, these upstream inhibition strategies have the potential to address cognitive impairment and neurological dysfunction in PDE, which can persist even with pyridoxine therapy.

### 2.9. Serine Deficiency

The most frequently reported cause of serine deficiency is 3-phosphoglycerate dehydrogenase (3-PGDH) deficiency (OMIM #601815), which affects the first step in the serine biosynthesis pathway [104,109]. Deficiency in phosphoserine aminotransferase (PSAT) (OMIM #610936), which catalyses the second step in the pathway, is a much rarer cause of serine deficiency and has only been reported in a few cases in the literature. The clinical features of serine deficiency may include seizures, microcephaly, hypertonia, and developmental delay. The suggested supplementation for infants with PSAT1 deficiency are serine (500 mg/kg/day) and glycine (200 mg/kg/day) [110].

### 2.10. Cystinuria

Cystinuria (OMIM #220100) is an autosomal recessive disorder with an incidence of 1 in 7000 that occurs due to pathogenic variants in SLC3A1 (2p21) or SLC7A9 (19q13.11) [111,112]. This results in the failure to absorb dibasic amino acids cystine, lysine, arginine, and ornithine by the proximal tubules and the intestinal tract. The main clinical feature of cystinuria is recurrent nephrolithiasis. The goal of treatment is to prevent stone formation or growth. This is achieved by increasing fluid intake, maintaining a moderate protein diet, urinary alkalinisation, and therapy with cystine-binding medications (alpha-mercaptopropionylglycine, tiopronin, and D-Penicillamine) [113,114]. Given that up to 70% of patients develop cystine stones or chronic kidney disease [115], follow-up with nephrology and urology is crucial in managing these patients (Table 6).

A first real-world pharmacovigilance analysis of tiopronin, using the FDA Adverse Event Reporting System (FAERS), showed that most safety reports involve children and are submitted by physicians. Previously unlabelled adverse events for tiopronin, including hyposmia, skin atrophy, and tongue discolouration, and frequent reports of incorrect paediatric dosing were detected [116]. These findings emphasise the need for strengthened caregiver and clinician education, standardised paediatric dosing frameworks, and focused long-term pharmacovigilance.

It has been hypothesised that continuous glucose exposure to cystine may trigger a Maillard reaction, preventing stone growth [117,118]. As such, sodium–glucose cotransporter 2 inhibitors (SGLT2) like dapagliflozin have been proposed as potential treatments for cystinuria. In one study, the off-label treatment of 10 patients with dapagliflozin reduced stone events compared to historical rates and resulted in few adverse events [118]. Other treatments are under early preclinical investigation, and include those aimed to reduce cysteine lithiasis such as 8-l-Cystinyl Bis(1,8-diazaspiro[4.5]decane) [119] and the antioxidant l-Ergothioneine [120], as well as transposon-mediated gene therapy [121] and AAV9-based gene therapy [122].

**Table 6:** Medical and dietetic management of basic amino acid metabolism or transport disorders.

Disorder	Treatment	Rationale/Mechanism	Dose	Monitoring
Cystinuria [123]	Potassium citrate	Urine alkalisation	Children: 60–80 mEq/1.73 m^2^/d Adults: 60–80 mEq/d TDS/QDS	Urine pH
	Penicillamine	Increases cystine solubility	Children: 20–30 mg/kg/d (max 4000 mg/d) Adults: 1–4 g/d TDS/QDS	Urine cystine excretion
	Tiopronin	Increases cystine solubility	Children: 15–40 mg/kg/d (max 1500 mg/d) Adults: 800–1500 mg/kg/d TDS	Urine cystine excretion
	Alpha-lipoic acid	Increases cystine solubility	Children: 30 mg/kg/d (max 1200 mg/d) Adults: 1200 mg/d BD	Urine cystine excretion
	Captopril	Increases cystine solubility	Children: 1.5–6 mg/kg/d (max 150 mg/d) Adults: 75–150 mg/d TDS	Urine cystine excretion
Lysinuric protein intolerance	Acute management [124]	Reduction in protein and caloric supplementation for preventing protein catabolism	Glucose infusion: 10% glucose (in cases of hyperglycaemia, consider adding insulin) L-arginine: 100–250 mg/kg/d IV Sodium phenylbutyrate: 450–600 mg/kg/d in patients <20 kg, 9.9–13.0 g/m^2^/d in larger patients Sodium benzoate: 100–250 mg/kg/d PO or IV +/− continuous haemodialysis +/− antibiotics (e.g., neomycin), lactulose, and/or lactobacillus preparation	Blood ammonia, amino acids in blood/urine, blood glucose
	Dietary: protein restriction, vitamin D, iron, zinc, and calcium supplementation, +/−medical foods, e.g., protein-free drinks	To prevent hyperammonaemia. Zinc, iron, calcium and vitamin D levels tend to be decreased.	Children: 0.8–1.5 g/kg/d protein intake Adults: 0.5–0.8 g/kg/d protein intake [125]	Amino acid (e.g., lysine, arginine, ornithine, glutamine) analysis in blood/urine; 25(OH)D, iron, zinc, calcium levels
	L-citrulline [124]	Reduces blood ammonia level, increases in dietary intake, reduction in hepatomegaly	100 mg/kg/d	Blood ammonia level, amino acids
	L-arginine [124]	Reduces blood ammonia level	120–380 mg/kg/d	Blood ammonia level, amino acids
	L-carnitine [124]	Secondary carnitine deficiency	20–50 mg/kg/d	Blood carnitine level, amino acids
	L-lysine [124]	Increases blood lysine levels	20–50 mg/kg/d	Blood lysine level, amino acids
	Nitrogen scavengers [124]	Decreases blood ammonia levels	Sodium phenylbutyrate: 450–600 mg/kg/d in patients weighing < 20 kg and 9.9–13.0 g/m^2^/d in larger patients Sodium benzoate: 100–250 mg/kg/d	Blood ammonia levels, plasma amino acids, electrolytes (Sodium)
	Other treatments [124]	Management of osteoporosis, short stature, hyperlipidaemia, nephritis, pulmonary alveolar proteinosis, and ESRD.	Vitamin D and bisphosphonate, GH injection, statins, ACE inhibitors, corticosteroids, whole lung lavage, GM-CSF, renal transplantation	As per clinical finding
Hartnup disease [126]	Nicotinamide	Management of dermatological and neurological complications.	50–300 mg PO [127]	
	High-protein diet	To compensate for amino acid loss	Individualised to patient	

### 2.11. Lysinuric Protein Intolerance

Lysinuric protein intolerance (LPI) (OMIM #222700) is caused by mutations in the gene coding for solute carrier family 7A member 7 (SLC7A7) located at chromosome 14q11.2, resulting in defective dibasic amino acid (lysine, arginine, ornithine) transport at the epithelial cells in the kidney and intestine [128]. Studies have shown an incidence rate of 1:60,000 in Finland and 1:57,000 in Japan [129]. The diagnosis is established by detecting the elevated 24 h urinary excretion of lysine with low plasma lysine. Most patients have episodes of hyperammonaemia, e.g., postprandially, due to a substrate deficiency and disruption of the urea cycle. Chronic kidney disease occurs in many patients, with 10% progressing to end-stage renal disease, which may be managed with dialysis and transplantation [130]. LPI may also result in chronic microcytic normochromic anaemia, proposed to result at least in part from reduced erythropoietin (EPO) production [131]. Many patients also develop progressive interstitial lung changes and subsequently life-threatening pulmonary alveolar proteinosis [132]. The diagnosis is confirmed through molecular genetic testing. Treatment is based on a protein-restricted diet and citrulline supplementation. Nitrogen scavengers are used to treat hyperammonaemia. L-arginine and lysine supplementation may also have a role in the treatment of LPI [133] (Table 6).

### 2.12. Hartnup Disease

Hartnup disease (OMIM #234500) is a rare, autosomal recessive disorder caused by pathogenic variants in the SLC6A19 gene (5p15.33), encoding the sodium-dependent neutral amino acid transporter B0AT1. This leads to the defective transport of neutral amino acids across epithelial cells in renal proximal tubules and the gastrointestinal tract [134,135]. Symptoms include pellagra-like skin eruptions, ataxia, spasticity, delayed motor development, hypotonia, and psychiatric symptoms [135,136,137]. Diagnosis is established by detecting hyperaminoaciduria. Confirmation relies upon mutation analysis. Treatment includes a high-protein diet and nicotinamide supplementation [126] (Table 6).

It has been shown that B0AT1 in the intestine functionally depends on forming a complex with angiotensin-converting enzyme 2 (ACE2) [138]. Mechanistic evidence shows that nine Hartnup-associated SLC6A19 missense variants cause the ER retention of B0AT1 and impair its trafficking to the plasma membrane, including some that also trap wild-type ACE2. The study concludes that the ER retention of mutated B0AT1 likely contributes to pathogenesis by impairing Na^+^-dependent neutral amino acid transport [139]. Therefore, the therapeutic modulation of ER protein-folding and exit pathways, such as through pharmacological chaperones and intracellular trafficking modulators, may be a mechanistically rational therapeutic strategy.

### 2.13. Glutaric Aciduria Type 1

Glutaric Aciduria type I (GA1) (OMIM #231670) is an autosomal recessive disorder of lysine, hydroxylysine, and tryptophan metabolism characterised by a deficiency of glutaryl-CoA dehydrogenase (EC 1.3.8.6), an enzyme encoded by the GCDH (OMIM #608801) gene, resulting in elevations of glutaric acid and 3-hydroxyglutaric acid (Figure 2b). It has an incidence of 1 in 100,000 live births, with a higher prevalence in Oji Cree natives, the Amish community, and Irish Travellers [140]. Neuroimaging features include a widening of the Sylvian fissures, a widening of the mesencephalic cisterns, and CSF space expansion anterior to the temporal lobes, collectively resulting in micro-mesencephalic macrocephaly [141]. The widening of the subarachnoid space may lead to a rupture of the bridging cortical veins, resulting in low-trauma subdural haematoma [142]. During the early years of life, acute striatal necrosis is one of the main causes of morbidity and mortality [143]. Secondary carnitine depletion is common in untreated GA1 patients [144,145]. Current treatment includes nutritional therapy with a lysine-free, tryptophan-reduced diet, in addition to arginine-enriched amino acid mixtures. The early implementation of an “unwell” regime, reducing or stopping natural protein intake temporarily and increasing calorie supply with an increased carnitine supplementation, together with the use of antipyretics, is crucial to reduce the risk of an acute metabolic and encephalopathic crisis with neurological sequelae. Lifelong carnitine supplementation is provided with the aim of inducing the elimination of toxic metabolites. An initial oral dosage of 100 mg L-carnitine/kg per day, divided into three doses, is commonly used [146,147], with the aim of maintaining high–normal reference range levels of free carnitine in plasma or in dried blood spots [148]; the dose may be doubled during crisis. Patients affected by dystonia may benefit from baclofen, trihexphenidyl, gabapentin, or even deep brain stimulation [140,148,149].

On the horizon is a novel substrate reduction AAV-microRNA therapy, which is targeting the enzyme α-aminoadipic semialdehyde synthase in the metabolic pathway of lysine [150], and an evolving gene therapy approach for glutaric aciduria type I was recently established in mice [151]. VGM-R02b is an AAV9-GCDH therapy in phase I clinical trials in China (NCT06217861) [152].

Another disorder of lysine metabolism is familial hyperlysinaemia, which includes two conditions: hyperlysinaemia type I (OMIM #238700) and hyperlysinaemia type II (OMIM #268700) [153]. Hyperlysinaemia type I is the most common form. It is caused by pathogenic variants in the AASS (OMIM #605113) gene, which provides instructions for the production of aminoadipic semialdehyde synthase (1.2.1.31) [154]. It is characterised by elevated concentrations of lysine in the plasma and the cerebrospinal fluid. Hyperlysinaemia is generally considered to be a benign metabolic condition. Clinical features may include psychomotor delay, hypotonia, and seizures. It remains unclear whether protein or lysine restriction is beneficial in symptomatic patients [155].

### 2.14. Hyperprolinaemia Type I and Type II

Hyperprolinaemia type I (HPI) (OMIM #239500) is caused by a deficiency in proline dehydrogenase (POX) (EC 1.5.5.2). HPI is characterised by increased plasma proline levels without an increase in the urinary excretion of ∆1-pyrroline-5-carboxylic acid (P5C) [156]. POX is encoded by the PRODH (OMIM #606810) gene located on chromosome 22q11, and, as such, its deficiency may occur because of contiguous gene deletion as part of DiGeorge syndrome [157]. Its clinical significance is not fully established, but may be associated with schizophrenia, autism, and seizures [156,158,159]. Hyperprolinaemia type II (HPII) (OMIM #239510) occurs due to the absence of the enzyme ∆-1-pyrroline-5-carboxylic acid dehydrogenase (EC 1.2.1.88), encoded by ALDH4A1, and is associated with higher proline levels than in HPI [160]. It typically results in drug-resistant but pyridoxine-sensitive seizures in the first year of life and, in the absence of timely treatment, may lead to developmental delay [160]. These seizures may occur because of the inactivation of vitamin B6 by accumulated Δ1-pyrroline-5-carboxylic acid, and therefore long-term vitamin B6 supplementation may prevent these seizures. Strict dietary therapy via the restriction of protein is not necessary, as it only results in a modest reduction in plasma proline and does not have an impact on clinical phenotype, and, in our centre, we advise to avoid protein excess. Antioxidants, such as vitamin C, may also have a minor role in treatment [160] (Table 7).

### 2.15. Glutamine Synthetase Deficiency

Glutamine synthetase deficiency (GSD) (OMIM #610015) is an extremely rare inborn error of glutamine metabolism caused by mutations in the GLUL gene (OMIM #138290). Clinical features include respiratory failure, encephalopathy, and brain malformations [166,167]. Laboratory findings include low levels of glutamine in plasma and cerebrospinal fluid (CSF). L-glutamine supplementation with the goal of glutamine normalisation may improve brain functioning. Therapy may be commenced at a low dose of 17 mg/kg/day, increasing slowly to higher doses of 1020 mg/kg/day, while monitoring plasma and CSF glutamine concentrations [168]. Gene therapy has not been specifically studied for GSD. However, the baculovirus-mediated restoration of glutamine synthetase has been shown to reduce hyperammonaemia in a chronic liver disease mouse model [169], offering proof-of-concept of the therapy’s feasibility.

### 2.16. Asparagine Synthetase Deficiency

Asparagine synthetase deficiency (ASD) (OMIM #615574) is an autosomal recessive disorder caused by mutations in ASNS (OMIM #108370) [170]. More than 20 cases have been reported [171]. Clinical features include microcephaly, developmental delay, seizures, axial hypotonia, and spastic quadriplegia. Laboratory findings include low plasma and cerebrospinal fluid (CSF) asparagine levels [172]. As of the time of this review, there are still no proven disease-modifying treatments for ASD, nor ones in clinical trials. Treatment is supportive, including antiepileptic medications and L-asparagine supplementation.

## 3. Discussion

Recent advancements in the treatment of IEAAMs have expanded therapeutic options beyond strict dietary management to include different pharmacologic agents, enzyme substitution, transplant options, and emerging genetic therapies in an overall more personalised therapeutic approach. Despite these developments, many conditions still require lifelong interventions and individualised treatment plans based on genotype, metabolic profile, and responsiveness to therapy. Clinical guidelines for the management of inborn errors of metabolism have been increasingly refined over time, incorporating emerging evidence and therapeutic advances to enhance consistency, efficacy, and patient outcomes in clinical practice.

The complete European guidelines on phenylketonuria diagnosis and treatment were published in 2017 [6] and later revised in 2025 [173]. The European Society for Phenylketonuria (ESPKU) guidelines have recommended a lifelong protein-restricted diet with an upper Phe target of 600 μmol/L for adult PKU patients [174]. High Phe levels in adulthood may directly affect mood and sustained attention, as demonstrated by neuropsychological testing during periods of Phe supplementation [175]. Sapropterin dihydrochloride, the synthetic form of BH4, was approved as the first pharmacological chaperone to correct the loss-of-function of the enzyme Phe hydroxylase (PAH) [176]. BH4 has a significant lowering effect on blood Phe concentration and can improve Phe tolerance with an acceptable safety profile [8,177]. There is still a lack of knowledge in predicting BH4 responsiveness, with at least half of those with PKU having either minimal or no response [178]. Other studies have reported BH4 responsiveness to range from 20% to 62% [22,179]. Suggested predictors of BH4 responsiveness include Phe levels at diagnosis, the Phe/Tyr ratio, Phe tolerance before BH4 treatment, and genotype [180]. In a study of 46 Italian PKU patients investigated for BH4 responsiveness, 17 patients were identified as BH4 responders [181]. The presence of at least one pathogenic variant with residual enzymatic activity was the best predictor of BH4 responsiveness, while the presence of two inactive alleles excluded responsiveness [181]. The assessment of BH4 responsiveness requires a pre-loading test and BH4 loading test. BH4 responders may be started on long-term treatment with BH4 at the initial dose of 10 mg/kg/day [181]. Pegvaliase is an enzyme substitution therapy for adults with PKU [10]. Pegvaliase is the first approved enzyme substitution therapy that can be considered for adult PKU patients who have failed existing management strategies [182]. Pegvaliase has a generally tolerable safety profile in adults with PKU [183,184]. An update of the web-based PKU guidelines for improving clinical outcomes and promoting the consistency of nutrition management for PKU patients receiving pegvaliase therapy has been published recently [185]. In adulthood, the goal of treatment is to maintain normal brain cognitive function and neuropsychological and social performance. A PKU-related health-related quality of life (HRQoL) questionnaire of patients and their families was developed in different subgroups of patients defined according to the severity of PKU, overall health status, and treatment with tetrahydrobiopterin (BH4). Data were collected and analysed from 253 parents and 306 patients, including 104 adults. It was shown that BH4 treatment was associated with better scores in all ages. This may reflect that a less restricted died, often made possible by a responsiveness to BH4, will have a positive impact on HRQoL in PKU patients [186]. A study by Bik-Multanowski and colleagues in 2008 of treatment noncompliant adults with PKU found an improvement of subjective well-being in patients with severe or moderate distress upon return to a recommended diet [187]. These studies highlight the importance of considering patient psychological and emotional well-being as part of the holistic treatment of IEAAMs such as PKU. Dawson et al. investigated the effect of Phe levels on reaction time [188]. Patients with PKU were split into three groups: off-diet (Phe > 1200 μmol/L), on-diet (Phe < 800 μmol/L), and maternal diet (Phe 100–400 μmol/L). Adults who discontinued the PKU low-Phe diet during adolescence were found to have slower reaction times than controls. Reaction times were measured before and after commencing a maternal PKU protein-restricted diet in 16 women who were contemplating pregnancies. Reaction times significantly improved as Phe levels were strictly controlled. The data show that the effects of Phe levels on reaction time are reversible [188]. Even well-controlled PKU has several subtler physical, cognitive, and behavioural consequences that have been recognised. In the healthy population, Tyr is considered a nonessential amino acid; however, in patients with PKU, it becomes essential. Tyr is converted into L-dopa, which serves as the precursor for the synthesis of catecholamines [189]. As such, it has been suggested that approximately 8% to 10% of the total protein calculated in the diet must come from Tyr [190]. One study [191] evaluated all randomised or quasi-randomised trials investigating the use of Tyr supplementation versus a placebo in people with PKU in addition to, or instead of, a Phe-restricted diet. In three trials reporting the results of a total of 56 participants, blood Tyr concentrations were significantly higher in the participants receiving Tyr supplements than those in the placebo group. No significant differences were found between any of the other outcomes measured. From the available evidence, no recommendations can be made about whether Tyr supplementation should be introduced into routine clinical practice [191].

The main aim of the various treatments of AKU used to date has been to achieve the symptomatic control of comorbidities such as arthritis and joint pain. A potential side effect of nitisinone is hypertyrosinaemia, potentially necessitating the dietary restriction of Tyr and Phe. In addition, long-term studies are needed to show the effectiveness of nitisinone in providing an adequate reduction in homogentisate to prevent the development of complications in AKU. The management of osteoporosis in AKU proves challenging due to various reasons, including the reduced reliability of dual energy X-ray absorptiometry scans due to extensive disc calcification, degenerative arthritis, or joint replacements [192,193]. In addition, fragility fractures may occur despite appropriate bisphosphonate therapy. It has therefore been recommended that osteoporosis in AKU should be initially treated with teriparatide and later with intravenous zoledronic acid [32]. Vitamin C is an antioxidant believed to reduce the conversion of HGA to benzoquinone acetate via oxidation, and one study highlighted that it serves as a cofactor for 4-hydroxyphenylpyruvate dioxygenase, which causes an increased HGA production [194].

While significant advances have been made in the treatment of MSUD, particularly in the context of dietary management, it remains a volatile and life-threatening illness. Liver transplantation, both from living and deceased donors, has been investigated as a potential treatment. Liver transplant from an unrelated deceased donor can restore 9–13% of whole-body BCKA metabolism. Over the intermediate term, living related donor transplant was shown to more effectively correct leucine and valine concentrations than deceased donor transplant. While neither form of transplantation provided an absolute protection from metabolic derangement, they may still offer a viable alternative or adjunct to dietary treatment [195].

In NKH, sodium benzoate can significantly reduce serum glycine levels, even when administering low doses of 53 mg sodium benzoate/kg body mass per day. However, a higher dose up to 240 mg/kg BM per day could not normalise CSF glycine in one study [196]. Sodium benzoate has an unpleasant taste and may cause itching, hyperactivity, and gastrointestinal discomfort. The accidental ingestion of toxic amounts of benzoate can occur when high doses are prescribed. However, no evidence of sodium benzoate toxicity was reported with the administration of high doses up to 470 mg/kg per day [196]. Even with good effort, numerous patients receiving high doses of benzoate had glycine levels that were over the specified target range, indicating that benzoate uptake is poor, especially in adult patients. In patients with attenuated disease, an early treatment with dextromethorphan and sodium benzoate appears to be effective in normalising plasma glycine. A combination of a ketogenic diet and low-dose sodium benzoate therapy was found to be more effective in reducing plasma glycine levels than high-dose benzoate alone in six infants [197]. Combining sodium benzoate with NMDA receptor inhibitors, such as dextromethorphan or ketamine, has been demonstrated to reduce seizures and enhance neurocognitive outcomes [198,199].

The International PDE Consortium released the first consensus guidelines for the diagnosis and treatment of PDE-ALDH7A1 in 2021 [103]. The successful treatment of PDE hinges on early diagnosis, appropriate pyridoxine supplementation, and diligent monitoring. Treatment with pyridoxine and lysine reduction therapies (LRTs) demonstrated a decrease in pipecolic acid, α-AASA, and delta-1-piperideine-6-carboxylate (Δ1-P6C) [200]. Some reports found a significant increase in developmental testing scores for this treatment [201].

Revised recommendations on the diagnosis and management of glutaric aciduria type I were published in 2023 [148]. An experienced interdisciplinary team should initiate and oversee metabolic treatment, which involves dietary modifications that include foods low in tryptophan and lysine, carnitine supplementation, and accelerated emergency care during acute episodes of intercurrent disease. However, there is usually little chance of averting irreversible harm if treatment is started after symptoms appear. The vigorous and prompt treatment of fever or illness is necessary. Using dextrose in conjunction with electrolyte-containing fluids at a rate of 6–10 mg/kg/min and ensuring that sufficient calories are supplied constitute the main components of aggressive therapy during acute illness. There is currently no consensus on the potential benefits of medical formulas high in arginine [202]. Levocarnitine scavenger therapy is aimed at reducing the build-up of toxic metabolites and correcting secondary carnitine depletion. Treating dystonia arising from striatal damage may involve standard therapies, including baclofen, benzodiazepines, and botulinum toxin. However, the effective management of dystonia remains challenging, and predicting which medications are likely to be effective in individual cases is difficult.

## 4. Looking Forward: Clinical Innovation and Unmet Needs

The expanding therapeutic landscape for IEAAMs provides opportunities to reconceptualise these disorders as potentially modifiable and, one day, curable. Building on the evidence summarised in this review, we have identified several overarching priorities for future research and clinical practice. A priority is the optimisation and individualisation of existing dietary and pharmacologic strategies. For many IEAAMs, diet remains the cornerstone of treatment. However, the evidence for the implementation of amino acid or protein restriction is often obtained from small cohort studies. The experience in PKU, where the liberalisation of low-Phe fruits and vegetables improved quality of life without compromising metabolic control, illustrates how careful analysis and real-world monitoring can refine dietary management and support more flexible regimens. Similar systematic work is needed in other IEAAMs through the establishment of a stronger evidence base.

A second major focus is the safe implementation of gene- and RNA-based therapies. Multiple proof-of-concept studies now demonstrate durable biochemical rescue in animal models of IEAAMs such as PKU, tyrosinaemia type I, and MSUD. Early-phase clinical programmes, such as mRNA-3705 for mut-type MMA or AAV-based PAH and GCDH gene therapy, are particularly promising. However, future work must focus on vector design, dose finding, immunogenicity, off-target effects, and the oncogenic potential of these therapies. At the same time, emerging biologics and small molecules aimed at substrate reduction, transporter modulation, protein stabilisation, or chaperoning will likely complement gene-based therapies. Examples include methionine gamma-lyase approaches in homocystinuria and SGLT2 inhibitors in cystinuria. Many of these agents act in the gut or circulation and may be particularly useful in patients who are not candidates for gene therapy.

A third area of unmet need is the prevention of irreversible neurological injury during acute metabolic decompensation. The current emergency protocols rightly emphasise the rapid promotion of anabolism, restriction of offending substrates, and removal of toxic metabolites through dialysis or alternative pathway activation. However, there is limited evidence on the optimal thresholds for dialysis initiation in MSUD or on standardised neurocritical care bundles to mitigate injury in GA1 and MMA.

Beyond biochemical control, holistic outcome measures must be incorporated. The existing data on HRQoL in PKU suggest that even modest dietary liberalisation or BH4 responsiveness can translate into meaningful psychosocial benefits for patients and families. As such, comparable work is needed across the IEAAM spectrum. Particular attention should also be paid to the transition from paediatric to adult services, as well as preconceptual and pregnancy care.

Finally, these advances raise important questions regarding health system organisation and equity of access. Many of the therapies discussed in this review, from specialised formulas to mRNA and gene therapies, are costly and often available only in high-resource settings. Future work must therefore engage with implementation science, cost-effectiveness analysis, and policy-level advocacy to ensure access to diverse populations.

## 5. Conclusions

In summary, IEAAMs represent a heterogeneous group of disorders in which timely diagnosis, metabolic control, and emergency management remain central to preventing irreversible organ damage and neurodevelopmental sequelae. The goal of the treatment of IEAAMs is to normalise the metabolic imbalance at a cellular level and in physiological fluids as much as possible by implementing dietary modifications and pharmacological treatments, along with patient monitoring and emergency treatment if necessary. However, despite these advances, many patients face limitations in achieving complete metabolic control, and the risk of long-term complications persists. An acute metabolic crisis poses ongoing challenges, with an unmet need for more targeted neuroprotective measures. Future treatment options include substrate reduction therapies, pathway inhibitors, and gene therapies that offer promising avenues for more definitive and/or personalised treatment. Robust long-term data on the optimal timing of therapies, as well as their safety and impact on quality of life, are still lacking. The future priority should therefore be a coordinated international effort aimed at establishing prospective studies and clinical trials that integrate metabolic, neurocognitive, and patient-reported outcomes. Incorporating emerging precision therapies within multidisciplinary rare disease networks, with an attention to pharmacogenetics, transition of care, and equitable access, will be essential for translating molecular innovation into tangible reductions in morbidity for patients living with IEAAMs globally.

## Figures and Tables

**Table 1:** An overview of the current and future landscape of IEAAMs.

Disorder	OMIM	Incidence/Prevalence	Affected Gene	Main Organs/Systems Involved	On-Label FDA-Approved Drugs	Ongoing Clinical Trials for Novel Drugs	Recent Preclinical Research
Phenylketonuria	261600	1 in 16,000 live births in USA; higher in Turkey and Ireland	PAH	CNS, skin	Sapropterin (2007) Pegvaliase (2018) Sepiapterin (2025)	JNT-517 (NCT06637514, NCT05781399) NGGT002 gene therapy (NCT06332807, NCT06061614)	HDAC6i reduces plasma Phe; GluN2B inhibition limits cognitive impairment.
Alkaptonuria	203500	Global prevalence of 1:250,000–1:1,000,000.	HGD	Musculoskeletal, heart, kidneys, sclera	Nitisinone (2025)	None	SAA1.1 allele as potential amyloidosis biomarker.
Tyrosinaemia type I	276700	1 in 100,000 live births; higher in Scandinavia and Québec	FAH	Liver, kidneys	Nitisinone (2002)	None	Lentiviral FAH expression in pigs; CRISPR deletion of HPD results in metabolic correction but increases HCC in mice. Genetically engineered Tyr-degrading *E. coli*.
Tyrosinaemia type II	276600	Incidence less than 1 in 250,000	TAT	Eyes, skin, CNS	None	None	None in scope of this article.
Tyrosinaemia type III	276710	Less than 20 cases reported	HPD	CNS	None	None	None in scope of this article.
Homocystinuria	236200 236250	Worldwide prevalence 0.8/100,000	CBS MTHFR	CNS, eyes, cardiovascular	Betaine (1996)	Pegtibatinase (HARMONY trial paused, may resume 2026)	SYNT-202 (methionine gamma-lyase). AAVrh.10-CBS gene therapy. Minicircle-based gene therapy.
Methylmalonic acidaemia	251000 251100 251110	1:50,000 to 1:360,000 live births	MMA MMUT MMAB	CNS, kidneys	Carglumic acid (2021)	mRNA-3705 (NCT04899310)	None in scope of article.
Maple syrup urine disease	608348 620698 620699	Incidence 1 in 185,000	BCKDHA BCKDHB DBT	CNS, liver, musculoskeletal	None	None	AAV8 gene therapy. AAV9 dual gene therapy. Lipid nanoparticles encapsulating mRNA.
Nonketotic hyperglycinaemia	605899	Incidence 1 in 76,000	GLDC AMT	CNS	None	None	AAV9-GLDC gene therapy.
Pyridoxine-dependent epilepsy	266100	Incidence 1 in 65,000	ALDH7A1 PNPO PLPBP	CNS	None	None	Targeting upstream AASS or lysine α-ketoglutarate reductase.
Cystinuria	220100	Incidence 1 in 7000	SLC3A1 SLC7A9	Kidneys	D-Penicillamine (1970) Tiopronin (1988)	None	SGLT2i through Maillard reaction. 8-l-Cystinyl Bis(1,8-diazaspiro[4.5]decane). l-Ergothioneine. Transposon-mediated gene therapy. AAV9 gene therapy.
Lysinuric protein intolerance	222700	Incidence 1:60,000 in Finland and 1:57,000 in Japan	SLC7A7	Kidneys, respiratory	None	None	Anaemia partly due to EPO deficiency.
Hartnup disease	234500	Incidence 1 in 15,000	SLC6A19	Skin, CNS	None	None	SLC6A19 variants may result in ER retention of B0AT1 and ACE2.
Glutaric aciduria type I	231670	Incidence 1 in 100,000; higher in Oji Cree, Amish, and Irish Travellers	GCDH	CNS	None	VGM-R02b (NCT06217861)	AAV-microRNA targeting AASS, AAV9-GCDH.
Serine deficiency	601815 610992	Not known; >50 reported cases	3-PGDH PSAT	CNS	None	None	None in scope of article.
Hyperprolinaemia type I	239500	Unknown	POX	Not adequately established, evidence for CNS	None	None	None in scope of article.
Hyperprolinaemia type II	239510	Unknown	ALDH4A1	CNS	None	None	None in scope of article.
Glutamine synthetase deficiency	610015	Unknown	GLUL	CNS	None	None	None specifically for GSD, but GS gene therapy has been successful in mice to reduce hyperammonaemia in liver disease.
Asparagine synthetase deficiency	615574	More than 20 cases reported	ASNS	CNS	None	None	None in scope of article.

**Table 7:** Medical and dietetic management of ornithine and proline disorders.

Disorder	Treatment	Rationale/Mechanism	Dose	Monitoring
Δ1-Pyrroline-5-carboxylate synthetase deficiency	Arginine	Increases arginine availability to brain; improvement of neurodevelopmental and metabolic parameters	150 mg/kg/d [161]	Amino acid analysis (proline, ornithine, arginine, citrulline), and ammonia levels
Hyperprolinaemia type I	Anti-epileptic medication and schizophrenia medication if required			
	Avoid protein excess	Reduce accumulation of proline or P5C		Plasma amino acids (proline)
Hyperprolinaemia type II	B6 supplementation	Avoid deficiency	e.g., 50–100 mg/day [162]	B6 levels
	Avoid protein excess	Reduce accumulation of proline or P5C		Plasma amino acids, urine organic acids
	Anti-epileptic medication and schizophrenia medication if required			
Ornithine δ-aminotransferase deficiency (gyrate atrophy)	Arginine-restricted diet with synthetic amino acid supplementation	Aim to decrease plasma ornithine levels and slow disease progression	10–35 g/d protein intake [163]	Ornithine and arginine levels
	Trial of B6, lysine, and creatine supplementation	B6—aims to stimulate residual enzyme activity; lysine—may increase kidney excretion of ornithine and arginine; creatine and precursors—to treat secondary creatine deficiency	B6: 100–1000 mg/d [163]	B6 and plasma amino acids; blood/urine creatine
Hyperornithinaemia–hyperammonaemia– homocitrullinuria	Acute management	Stop protein intake for 24 h and commence IV 10% glucose (plus electrolytes); arginine +/− citrulline supplementation; Ammonia scavengers (sodium benzoate and sodium phenylbutyrate); +/−haemodialysis (if neurological status is deteriorating)	Glucose dose at appropriate dose to prevent catabolism; sodium benzoate: 250 mg/kg bolus (90–120 min), then maintenance 250–500 mg/kg/d (>20 kg, 5.5 g/m^2^/d); sodium phenylbutyrate: 250 mg/kg bolus (90–120 min), then 250–500 mg/kg/d as maintenance [164]	Blood ammonia levels, blood glucose
	Long-term management	Protein-restricted diet with citrulline or arginine (+/−sodium benzoate or sodium phenylbutyrate)	Protein restriction individualised to patient Sodium benzoate: ≤250 mg/kg/d Sodium phenylbutyrate: <20 kg ≤250 mg/kg/d, >20 kg 5 g/m^2^/d L-citrulline: 100–200 mg/kg/d L-arginine: <20 kg 100–200 mg/kg/d, >20 kg 2.5–6 g/m^2^/d [164]	Blood ammonia levels, plasma amino acids, urinary orotic acid
	Creatine supplementation [165] (if plasma creatine levels low)	To treat secondary creatine deficiency	Dosed according to degree of creatine deficiency	Plasma creatinine levels, blood/urine creatine

## Data Availability

No new data were created or analysed in this study.

## Abbreviations

The following abbreviations are used in this manuscript:

5-HIAA	5-Hydroxyindoleacetic acid
AASS	Aminoadipic semialdehyde synthase
AAV	Adeno-associated virus
AAV8	Adeno-associated virus serotype 8
ACE	Angiotensin-converting enzyme
AEDs	Anti-epileptic drugs
AKU	Alkaptonuria
AMT	Aminomethyltransferase
ASD	Asparagine synthetase deficiency
ASNS	Asparagine synthetase gene
BCAAs	Branched-chain amino acids
BCKD	Branched-chain ketoacid dehydrogenase
BCKDC	Branched-chain α-ketoacid dehydrogenase complex
BCKDH	Branched-chain ketoacid dehydrogenase
BD	Twice daily
BH4	Tetrahydrobiopterin
BHMT	Betaine-homocysteine methyltransferase
BTMs	Bone turnover markers
C3	Propionylcarnitine
CBS	Cystathionine-β-synthase
CNS	Central nervous system
CSF	Cerebrospinal fluid
CT	Computed tomography
CYP2D6	Cytochrome P450 2D6
CYP3A4	Cytochrome P450 3A4
CYPUGT	Cytochrome P450 / UGT
DNAJC12	DnaJ heat shock protein family (Hsp40) member C12
EC	Enzyme Commission
ESPFKU	European Society for Phenylketonuria
ESRD	End-stage renal disease
FAA	Fumarylacetoacetate
FAH	Fumarylacetoacetate hydrolase
GA1	Glutaric aciduria type 1
GCDH	Glutaryl-CoA dehydrogenase
GH	Growth hormone
GLDC	Glycine decarboxylase
GLUL	Glutamate–ammonia ligase (glutamine synthetase) gene
GM-CSF	Granulocyte macrophage colony-stimulating factor
GSD	Glutamine synthetase deficiency
HCU	Homocystinuria
HGA	Homogentisic acid
HGD	Homogentisate 1,2-dioxygenase
HRT	Hormone replacement therapy
HRQoL	Health-related quality of life
HPD	4-Hydroxyphenylpyruvate dioxygenase
HVA	Homovanillic acid
IEAAMs	Inborn errors of amino acid metabolism
IV	Intravenous
L-DOPA	L-3,4-dihydroxyphenylalanine
LNAAs	Large Neutral Amino Acids
LP	Lumbar puncture
LPI	Lysinuric protein intolerance
LRTs	Lysine reduction therapies
MAA	Maleylacetoacetate
MAAI	Maleylacetoacetate isomerase
MAT	Methionine S-adenosyltransferase
MMA	Methylmalonic acidaemia
MMAA	Methylmalonic acidaemia cblA type
MMAB	Methylmalonic acidaemia cblB type
MMUT	Methylmalonyl-CoA mutase
MS	Methionine synthase
MSUD	Maple syrup urine disease
MTHFR	Methylenetetrahydrofolate reductase
NaCl	Sodium chloride
NG	Nasogastric
NKH	Nonketotic hyperglycinaemia
NMDA	N-methyl-D-aspartate
NTBC	Nitisinone (2-(2-nitro-4-trifluoromethylbenzyl)-1,3-cyclohexanedione)
OMIM	Online Mendelian Inheritance in Man
PAH	Phenylalanine hydroxylase
PAL	Phenylalanine ammonia lyase
PDE	Pyridoxine-dependent epilepsy
Phe	Phenylalanine
PKA	Protein kinase A
PKC	Protein kinase C
PKU	Phenylketonuria
PO	By mouth
PRODH	Proline dehydrogenase
QDS	Four times daily
SAH	S-adenosylhomocysteine
SAHH	S-adenosylhomocysteine hydrolase
SAM	S-adenosyl-L-methionine
SC	Subcutaneous
TAT	Tyrosine aminotransferase
TDS	Three times daily
THF	Tetrahydrofolate
tHcy	Total homocysteine
Tyr	Tyrosine
WBC	White blood cell
